# Society Meetings

**Published:** 1916-09

**Authors:** 


### SOCIETY MEETINGS

Brief reports and announcements of meetings of societies of Western New York. and those of general scope, are requested from Secretaries. Copy should be on hand the fifteenth of the month. Full transactions will be published at cost of composition.

**DOCTOR: Is your Society properly represented here? If not, please bring our standing announcement to the attention of the members.**

**Medical Society of the United States.** Organization Officers—A. H. Ohmann-Dumesnil, St. Louis, President; George Howard Thompson, St. Louis, Secretary; Emory Lanphear, St. Louis, Treasurer.

Vice-Presidents—Carl Keller, Honolulu; Nobel Younkin, Frankfort, Ind.; Oscar J. Fullerton, Waterloo, Ia.; William F. Waugh, Muskegon, Mich.; Bruno F. J. Getzlaff, Sutton, Neb.; Geo. L. Servos, M. D., Reno, Nev.; J. Necurvus Pyle, Mineral Wells, Tex.; C. A. Bryce, Richmond, Va.; Josef Francois Replogle, Wyoming.

The Annual Meeting of 1916 will be held in the Planters' Hotel, St. Louis, Mo., October 3d, 4th and 5th.

Organization—The Medical Society of the United States has been organized for the purpose of founding a great National Society which shall include all reputable members of the medical profession who have not (for one reason or another) affiliated themselves with the American Medical Association, or its branches, as well as members of that great body who have become disgusted with its management under present control. Graduates of homeopathic and eclectic colleges are admitted upon the same terms as those of the so-called "regular" schools. Its scheme of organization will be noted below.

Objects—The objects of this association shall be the improvement of the status of the Medical Profession of the United States through meetings, publications, lectures, etc., the promotion of the Public Health by means of improving sanitation and instructing the people in matters of Hygiene, Eugenics and Social Economy; to secure uniform examination and registration in the various States of the Union, together with more practical methods of reciprocity between State Boards of Health, and to issue to its members Certificates of

Membership which shall be of value in securing reciprocity, and to promote the interests of the Medical Profession of the United States in regulating, so far as possible by its influence, the conduct of medical schools, hospitals and journals, and to establish and maintain a Home for Aged Physicians to be supported by voluntary contributions from members of the association.

Location—While the membership of this Society may include reputable physicians of the entire United States, its territories and colonies, the general offices shall be located in the city of St. Louis, Missouri.

Management—The corporate affairs of this Society shall be managed by a Board of Directors composed of five members of the Society, who shall have power to engage and appoint employees (such as stenographers, clerks, editors and their assistants) and to discharge them, to carry on the financial affairs of the corporation—subject to the approval of the entire Society in annual meeting assembled. This Board of Directors shall be elected at the annual meetings of the Society, wherever held, and shall be subject to the instructions of the Society in such annual meeting assembled, in the matter of the selection of the Officers of the Society, who, with the exception of the President and Vice-Presidents, shall be the proper officers of the Board as well as of the Society. The Board shall have the right to select its own chairman.

Officers—The Officers of the corporation shall be a President, a Vice-President for each State, Territory and Colony of the United States, a Secretary, a Treasurer and a Manager (the last named of which may also hold any other office), whose respective duties shall be defined in the By-Laws to be adopted hereafter by the Board of Directors.

Powers—This corporation shall have the power to formulate rules and regulations for the conduct of its members, and to enforce them to the extent of expulsion of such members as shall be proven guilty of violation of them; shall have the right to establish branches in such cities and counties throughout the United States and its territories and colonies as the Board of Directors may deem best for the welfare of the Society; shall be empowered to hold property in its corporate name, to buy and sell such property, to publish and print periodicals, brochures, reprints, reports, transactions, etc., concerning its own affairs and the Science of Medicine and allied subjects, to accept bequests and donations; to own and operate printing establishments and employ such help as may be necessary therefor, and to conduct any and all such businesses as a natural man might wish to conduct.

Preliminary Program:

Detection of Monolateral Malingerers of Hearing, with Demonstration of Instrument, Carl B. Wagner, M. D., Chicago, Ill.

Electro-Therapeutics as a Specialty for the General Practician, Homer C. Bennett, M. D., M. E., Lima, Ohio, Secretary National College of Electro-Therapeutics.

The Internal Secretion from the Testicle, Winfield S. Hall, M. D., Ph. D., Chicago, Ill., Professor of Physiology in Northwestern University.

Some of the Uses of Electricity in Ophthalmic Practice, W. Franklin Coleman, M. D., M. R. C. S., Eng., Chicago, Ill., Professor Ophthalmology in the Post-Graduate Illinois Medical School.

Subject Unannounced, C. S. Neiswanger, M. D., Chicago, Ill., Professor of Electro-Therapeutics in the Post-Graduate Medical School of Chicago.

Why the Brain Has Little to do with Memory, Perception or Thinking, Leonard K. Hirschberg, A. B., M. A., M. D., Baltimore, Md., Chief of Clinic of Nervous Diseases Baltimore City Hospital, Instructor at Johns Hopkins University.

Significance of Remissions in Paretic Cases, R. Harvey Cook, M. D., Oxford, Ohio, Physician in Chief to Oxford Retreat.

New Procedures in the Field of Dermatology and Syphilis, William S. Gottheil, M. D., New York City, Professor of Dermatology and Syphilography in Fordham University Medical School.

Possibilities and Limitations of Radium, Evan O'Neill Kane, M. D., Kane, Pa., Surgeon in Chief to Kane Summit Hospital.

Subject Unannounced, J. C. Tritch, A. M., M. D., Findlay, Ohio, Gynecologist to Tritch's Home and Hospital.

Some Tumors of the Kidney with Presentation of Specimens, Emory Lanphear, M. D., Ph. D., LL. D., St. Louis, Mo., Chief Surgeon to the German Hospital.

Significance of Bladder-Symptoms in Gynecologic Practice, W. A. Newman Dorland, A. M., M. D., Chicago, Ill., Professor of Obstetrics, Loyola University.

Infectious Embolism: A Factor in Chronic Ailments and Convalescence, John Aulde, M. D., Philadelphia, Pa., Medical Director of Arden Health Institute.

Subject Unannounced, Finley Ellingwood, M. D., Chicago, Ill., Editor Ellingwood's Therapeutist.

Report on Some Original Investigations, Thomas H. Kelley, M. D., Chicago, Ill., Professor of Surgery in Loyola University.

President's Address: Some Observations and Reflections on Medical Ethics, A. H. Ohmann-Dumesnil, A. M., M. D., Ph. D., St. Louis, Mo., formerly Professor of Dermatology in the Marion-Sims College of Medicine.

Rectal Fistulae, Charles J. Drueck, M. D., Chicago, Ill.

Subject Unannounced, Adam Bode, M. D., St. Louis, Mo., Associate Gynecologist to the German Hospital.

Subject Unannounced, Emanuel F. Oehler, M. D., Ph. B., St. Louis, Mo., Associate Surgeon to the German Hospital.

Observations on the Allbe Operation for Tuberculosis of the Spine, F. L. Morse, M. D., St. Louis, Mo., formerly Professor of Genito-Urinary Surgery in the American Medical College.

Note: This announcement is accompanied with a request for the expression of an opinion. In general, we would not oppose any medical organization of proper purpose. In this instance, we reprecate, on principle, any action tending to divide the profession. The medical history of New York State has too recently illustrated the practical disadvantages of such a division. At the same time, we appreciate the causes of this movement. While we do not feel that either the A. M. A. as a body or its officials have been guilty of wrong doing, there is a widely diffused sentiment that it is too strongly and closely governed, and that such an organization, for an association as for a people involves potential dangers. Many believe that the state and county branches of the A. M. A. should be allowed greater elasticity of local organization, to conform to local conditions or desires; that it should be possible for every member to express opinions and to vote upon issues arising, that in short, there should be an average between the governed and the governing; that the official organization journals should offer the privilege of free discussion. Of course, these criticisms might be supplemented with a repetition of specific complaints, but we are sceptic as to the evils charged in the latter and we prefer to deal only with general matters of policy. But, we feel that the correction of the methods of the A. M. A. should be freed from personal bitterness and should take place within that body, in accordance with the real wishes of its membership. Meantime, it must be admitted that the strong central government of the A. M. A. has made it a powerful body, has immensely increased its scientific standards, has brought about many ethical improvements and has given a quid pro quo to its members. The movement to organize a rival society should, we believe, be turned in the proper direction to retain professional unity and to secure professional harmony and satisfaction.

---

**The Gross Medical Club.** The regular meeting was held on Friday, August 18th, at Alden, being entertained by Dr. B.

J. Gipple, Pres. W. Warren W. Britt occupying the chair.

A very interesting paper, Peritonsilar abscess, by Dr. Edgar Forsythe in which he reported a case in which infection extended to the brain resulting in abscess formation, was discussed by those present.

Dr. Arthur G. Bennett brought out that if adrenilin was added to cocaine when injecting into inflammatory tissue it would be absorbed—cocaine is not absorbed by inflammatory tissue if used alone.

Dr. Gipple reported the case of a child aged 12 who suddenly puffed; swelled all over the body to an extent the face was distorted almost out of recognition: breathing was not interfered with. He gave the child small doses of adrenilin and he recovered after a few hours. The general concensus of opinion was that this was a case of angeo neurotic oedema.

Dr. Rooker a case of headache, which he believed to be migraine and with periodical attacks had lasted for 18 years, nothing seemed to relieve, only rest in bed for two or three days seemed to have any effect. Patient was finally sent to nose and throat specialist who found involvement of the ethymoidal sinuses, these were operated on and there has been no headache in three months.

Dr. Bennett after being informed there was no eye or other involvement referrable to the brain, questioned the opinion of migrane, and recited the history of a case in which by eye examination the conditions of this kind are proven, and that when eye and other involvement is not present the condition is not migraine, but due to some condition of the nose. He minutely described the prodromal attack, calling attention to hemiopia as being about the first symptom present, the rest following in a short time. He remarked that the fearful headache was probably the most distressing, the one the patient complained of most and the one they felt the most interest in, as to relief. He believes the headache is due to first a spasm of certain vessels which gradually relax until a whole area may be involved, and if on the left side there will be more or less interference with speech. In one case of which he had a personal knowledge of for years, after careful study and the use of various preparations he had found that if when vision became sort of obscured and other of the premonitory symptoms present a vaso dilator be given at once (he has found that whisky is the best) very little if any headache will follow, in fact many times he has prevented it entirely.

Dr. Dowd, a case of a man that when erection occurred the penis bent upwards in the shape of a U, to such an extent that the glans touched the abdomen making intercourse impossible, this man was impotent. In these conditions something is always found blocking the Corpora cavernosa, it may be due to a cicatrix following an internal urethrotomy, a tumor or injury. In this case a firm mass could be felt at the root, cartilagenous in character. No internal urethrotomy had ever been performed, nor was stricture found. The patient having had syphilis, the condition was undoubtedly gumata, antisyphilitic treatment was ordered. Another case was that of a woman married about six years, she had never been pregnant, not missed a period. Consulted the reporter for what I diagnosed as neurasthenia. There was obstinate constipation, profuse leucorrhoea, no appetite and she was losing flesh. No evidence, except a catarrhal endometritis was found from vaginal examination. The urine showed perfect, except an index about 80% minus. Comp. Phos. Tonic (Dowd) M 25, res. pod. gr. 1-10 (for constipation) in glycerine for one drachm was ordered. This patient was not seen again until two or three days ago when she came to the office with her sister. She spoke of how well she felt, had gained much in weight, but no menses for three months: all symptoms of pregnancy were present. This is the fifth similar case occurring in my practice, yet in none of them was the condition spoken of, yet all courted pregnancy. We know that out of every six cases of sterility, the woman is at fault five times and that the most common cause is a granular endometritis, yet all will not submit to curetment. My only explanation is a dormant condition of the ovaries, and as the very life of the ovum is phosphorus and the ovaries directly under control of the brain and nervous system, it is only a suggestion—in sterility get the Phosphatic Index.

Dr. Phelps, a case of a butcher, who after skinning two head of cattle that had died, later proving to be from anthrax, contracted this disease. Two swellings occurred on thumb, looking like carbuncles. At first there was no fever, but it suddenly rose to 102 F, patient becoming slightly delirious. The swellings were both removed, examination showed them to be anthrax: he made a good recovery.

Dr. H. B. Deegan of N. Tonawanda and A. M. Rooker of Niagara Falls were elected to membership.

---

**New York and New England Association of Railway Surgeons.** The twenty-sixth annual session will be held at the Hotel McAlpin, New York City, on Wednesday, October 18, 1916. A very interesting and attractive program has been arranged. Dr. Wm. S. Bainbridge will deliver the "Address in Surgery," on the Cancer Problem. Railway surgeons, attorneys and officials and all members of the medical profession are cordially invited to attend. Dr. D. H. Lake, President, Kingston, Pa.; Dr. George Chaffee, Corresponding Secretary, Little Meadows, Pa.

---

**The American Assn. for Study and Prevention of Infant Mortality** will hold its seventh annual meeting at Milwaukee, Oct. 19-24. Technical, sociologic and popular papers will be presented, various affiliated organizations will report by committee and conferences will be held. An exhibit will be made. Those interested are invited to attend or to become members. Life membership is $200, sustaining $25 annually, contributing $10, active $3. Affiliated societies may be enrolled at $5 annually. Checks should be sent to the Treasurer, Austin McLanahan, 1211 Cathedral St., Baltimore. Portable exhibits can be secured for local campaigns on application.

---

**The Elmira Clinical Society** held its regular meeting, July 25, at the residence of Dr. Wm. Brady, who presented a paper on Mechanic Technic.

